# A comparative study of the quality differences and seasonal dynamics of flavonoids between the aerial parts and roots of *Scutellaria barbata*

**DOI:** 10.3389/fpls.2024.1497664

**Published:** 2024-12-02

**Authors:** Yijie Cheng, Wenxin Cao, Ru Guo, Ruihuan Chen, Xiaofan Li, Da Qian, Jingyuan Xu

**Affiliations:** ^1^ Pharmacy Department, Changshu Hospital Affiliated to Soochow University, Changshu No.1 People’s Hospital, Changshu, China; ^2^ Central Laboratory, Changshu Hospital Affiliated to Soochow University, Changshu No.1 People’s Hospital, Changshu, China; ^3^ School of Biology and Food Engineering, Changshu Institute of Technology, Changshu, China; ^4^ Suzhou Qifan Agricultural Technology Co., Ltd, Changshu, China

**Keywords:** *Scutellaria barbata*, quality differences, spatial distribution, seasonal dynamics, flavonoids

## Abstract

**Introduction:**

*Scutellaria barbata* D. Don is a widely cultivated Chinese herbal medicine known for its medicinal properties. However, differences in the spatial distribution of metabolites, accumulation patterns of flavonoids, and pharmacological activities between the aerial parts and roots of *S. barbata* still remain unclear, posing challenges for its standardized cultivation and quality control. This study aimed to elucidate the quality differences between these plant parts and clarify their seasonal variations.

**Methods:**

The chemical profiles were qualitatively analyzed by UPLC-QTOF-MS/MS. The accumulation patterns of total flavonoids, scutellarin and baicalin in different parts of *S. barbata* were quantitatively analyzed by UV and HPLC respectively. The differences of pharmacological efficacy were evaluated by antioxidant assays and CCK-8 assay.

**Results:**

In this research, there were 46 compounds identified in *S. barbata* that included 44 flavonoids. The aerial parts primarily accumulate flavonoids with 4′-hydroxyl group, while the root mainly accumulate flavonoids without this group. Additionally, the accumulation and variation of flavonoid components were seasonally dependent, with the aerial parts reaching peak content in spring during vigorous vegetative growth and the roots accumulating most flavonoids in autumn. The extracts from both parts exhibited antioxidant activity and inhibitory effects on cancer cell proliferation, with notable differences between them.

**Discussion:**

This study provides valuable insights into the quality differences and seasonal dynamics of the different parts of *S. barbata*, offering a reference for standardized harvesting and quality control.

## 1 Introduction

The therapeutic effects of medicinal plants are primarily attributed to the endogenous bioactive ingredients, most of which are secondary metabolites (Rogerio et al., 2010; Chen et al., 2024). The secondary metabolites in medicinal plants are diverse, accumulating in specific organs and exhibiting distinct distribution patterns. For instance, in *Astragalus membranaceus*, dihydroflavones, isoflavones, and flavones are predominantly found in the aerial parts, whereas saponins are primarily concentrated in the roots (Li et al., 2019; Gao et al., 2024). In addition, the accumulation of active ingredients in medicinal plants varies with the growth and development of the plants. For example, the young leaves of *Mitragyna speciosa* (Korth.) Havil. contain significantly higher levels of medicinally beneficial alkaloids, which decrease as the leaves mature (Kong et al., 2017; Veeramohan et al., 2023). Flavonoids, as active ingredients in many species of medicinal plants, exhibit organ-specific distribution, and their accumulation varies with the plant’s development (del Baño et al., 2004; Xu et al., 2018b; Bouyahya et al., 2022; Ali Reza et al., 2023). Therefore, a comprehensive study of the distribution differences and accumulation patterns of flavonoids in medicinal plants can provide valuable insights for selecting medicinal parts and improving the quality control of medicinal materials.

*Scutellaria barbata* is a perennial herb belonging to the genus *Scutellaria* in the family Lamiaceae. Its dried whole herb, known as Ban-zhi-lian (BZL) in traditional Chinese medicine, is widely used in clinical practice. It is recognized for its properties of expelling toxins, cooling, dispersing blood stasis, and promoting diuresis. Clinically, it is used to treat throat swelling and pain, injuries from falls, and bite wounds from snakes or insects (Chinese Pharmacopoeia Commission, 2020). Pharmaceutical research indicates that BZL extract exhibits a range of activities, including anticancer, anti-inflammatory, and antibacterial effects (Sato et al., 2000; Xu et al., 2013; Wu et al., 2022). BZL extract is rich in flavonoids, and researchers have currently isolated more than 50 flavonoid compounds with diverse structures from the extract (Wang et al., 2020). Most of the flavonoids isolated from BZL exhibit significant bioactivity. For instance, scutellarin can inhibit the proliferation of breast cancer stem cells, while baicalin improves insulin resistance and regulates liver glucose metabolism (Xu et al., 2018a; Ma et al., 2024). Therefore, flavonoids are used as one of the key criteria for the quality evaluation of BZL in the Chinese Pharmacopoeia and other related studies (Chinese Pharmacopoeia Commission, 2020).

As the demand for BZL continues to increase year by year, the BZL currently available on the market is primarily sourced from artificial cultivation. Because the aboveground parts of *S. barbata* can regreen multiple times a year, the aboveground parts of artificially cultivated *S. barbata* are often harvested several times annually for use as medicinal materials. In addition, during artificial cultivation, the harvested parts of *S. barbata* are often inconsistent across different batches. For example, some batches only contain the aerial parts, while others contain both the aboveground parts and roots. Previous studies have shown that the harvest time can significantly impact the quality of Chinese medicinal materials. The types and accumulation of active ingredients also vary greatly among different plant organs (Zeng et al., 2017; Hazrati et al., 2024). Therefore, non-standard harvesting methods may result in quality differences between batches of BZL. To our knowledge, research on the distribution and accumulation patterns of flavonoids in the aerial parts and roots of *S. barbata* is lacking. Additionally, there is a lack of comprehensive bioactivity assessment for different parts of *S. barbata*. This gap hinders the effective quality control of *S. barbata* through standardized cultivation and harvesting practices.

This study first identified the chemical components of the aerial parts and roots of *S. barbata* using high-performance liquid chromatography (HPLC)–quadrupole time-of-flight (QTOF)–tandem mass spectrometry (MS/MS) to compare the chemical profiles of these two parts. After that, the metabolic change pattern of the total flavonoids and the key active flavonoids in both parts were investigated. Building on these findings, the study further detected the antioxidant properties and cancer cell proliferation inhibition of the aerial part extract and root extracts to gain preliminary insights into their quality differences. These insights provide a theoretical foundation for the standardized harvesting and quality control of artificially cultivated *S. barbata*.

## 2 Materials and methods

### 2.1 Plant materials

From March 9 to December 14, 2023, the entire *S. barbata* was collected biweekly from the Qi Fan Agricultural Technology Co., Ltd., planting base. The collected samples were washed and divided into aerial parts (mainly leaves and stems) and roots. These samples were then dried according to the methods outlined in the Chinese Pharmacopoeia (Chinese Pharmacopoeia Commission, 2020).

### 2.2 Chemicals and reagents

Baicalin and scutellarin, the reference standards, were obtained from Baoji Herbest Bio-Tech Co., Ltd. (Shannxi, China). The purity of each compound was determined to be over 98% through the HPLC analysis. Methanol for chromatography was obtained from Merck (Darmstadt, Germany). Chromatography-grade water was obtained from Watsons (Hong Kong, China). All other reagents were of analytical grade and obtained from Nanjing Chemical Regents Co., Ltd. (Nanjing, China).

### 2.3 Chemical profile qualitative analysis by UHPLC–QTOF–MS/MS

Chromatographic separation was performed using a Waters ACQUITY UPLC (Waters, Milford, MA, USA). An ACQUITY UPLC BEH-C18 column (2.1 × 150 mm, 1.7 μm; Waters, Wexford, Ireland) was utilized for analysis at 30°C. The mobile phase for chromatographic separation consisted of a mixture of 0.1% formic acid–water (A) and methanol (B). The flow rate was 0.3 mL/min, with the following gradient conditions: 0–20 min, 5%–25% (B); 20–25 min, 25%–30% (B); 25–35 min, 30%–95%; 35–37 min, 95%–95%; and 37–40 min, 95%–5%. The injection volume was 5 μL. Qualitative analysis was conducted using the AB SCIEX Triple TOF 5600 system (AB SCIEX Technologies, Redwood City, CA, USA) in both negative and positive ion modes. The mass range was set from 100 to 2,000 *m*/*z*, and the electrospray ionization temperature was set to 50°C. The nebulizer gas pressure and ion spray voltage were 60 psi and 4.5 kV, respectively. The collision energy was −35 V for negative ion mode and 40 V for positive ion mode.

### 2.4 Quantitative analysis of total flavonoids by UV spectrophotometry

The standard curve of scutellarin and baicalin was established according to the Chinese Pharmacopoeia (2020 edition) for the quantitative analysis of total flavonoids in the aerial parts and roots of *S. barbata*, respectively. For sample preparation, approximately 1 g of powdered aerial parts of *S. barbata* (sieved through a No. 3 sieve) was placed in a Soxhlet extractor and extracted with petroleum ether (60°C–90°C) at a 1:100 material-to-liquid ratio. The mixture was refluxed at 82°C until colorless, after which the ether solution was discarded, and the residue was extracted with methanol until colorless. The extract was diluted to 100 mL with methanol, followed by further dilution of 1 mL of the solution to 50 mL. The absorbance was measured using an ultraviolet (UV) spectrophotometer at 335 nm. Root samples were directly extracted with methanol without prior treatment with petroleum ether, following the same procedure as for the aerial parts. The absorbance was measured at 278 nm.

### 2.5 Quantitative analysis of flavonoids by high-performance liquid chromatography

An HPLC method was developed to quantify scutellarin in the aerial parts and baicalin in the roots of *S. barbata*. All samples were analyzed using an Agilent Series 1260 LC system (Agilent Technologies, Cambridge, MA, USA). Chromatographic separation was performed with a mobile phase consisting of 0.1% formic acid in water (A) and methanol (B) under the following gradient conditions: 0–5 min, 15%–25% (B); 5–10 min, 25%–30% (B); 10–20 min, 30%–40% (B); 20–30 min, 40%–40% (B); 30–40 min, 40%–55% (B); 40–55 min, 55%–70% (B); 55–65 min, 70%–95% (B); 65–70 min, 95%–95% (B); 70–75 min, 95%–15% (B); and 75–78 min, 15%–15% (B). The detection wavelengths were set at 335 nm for the aerial parts and 278 nm for the roots. The column temperature was kept at 25°C, with a flow rate of 1 mL/min and an injection volume of 20 μL.

### 2.6 Method validation

Method validation was conducted following our previous research with slight modifications (Xu et al., 2018b). The validation process included analysis of the linear regression curve, limit of detection (LOD), limit of quantification (LOQ), repeatability, intra-day and inter-day stability, and recovery. Calibration curves were constructed using a series of standard solutions at appropriate concentrations. The LOD and LOQ were determined based on signal-to-noise (S/N) ratios of 3 and 10, respectively.

Precision was evaluated by analyzing standard solutions in six replicates for both intra-day and inter-day variability. To assess repeatability, six different sample solutions were prepared from the same root and aerial part samples collected on April 20, and the results were expressed as relative standard deviation (RSD). Stability was assessed by storing the sample solutions at 25°C and analyzing them at 0 hours, 2 hours, 4 hours, 6 hours, 8 hours, 12 hours, 24 hours, 48 hours, and 72 hours. For recovery validation, six replicates of root samples (collected on April 20) were added with baicalin for extraction and recovery assessment, while six replicates of aerial part samples (collected on April 20) were added with scutellarin. The results of method validation for HPLC and UV spectrophotometry are shown in **Supplementary Table S1**. The calibration curves are presented in **Supplementary Figure S1**.

### 2.7 Antioxidant assays

#### 2.7.1 ABTS scavenging assay

The ABTS scavenging assay was performed to evaluate the antioxidant activity, following a previously reported method with modifications (Cheng et al., 2022). The ABTS stock solution was prepared and diluted to achieve an absorbance of approximately 0.7 at 734 nm. The BZL extracts were mixed with the diluted ABTS solution and incubated in the dark at 30°C for 30 min. The absorbance of the mixture was measured at 734 nm to determine the antioxidant activity. The ABTS radical scavenging activity was calculated as follows:


$$\begin{array}{c} ABTS\;radical\;scavenging\;activity\;\left(\%\right)\\ =\frac{A0-\left(A1-A2\right)}{A0}\;\times \;100\% \end{array}$$

where A_1_, A_0_, and A_2_ are the absorbance values of the samples [or L-Ascorbic acid (Vc)], the control, and the blank without ABTS, respectively.

#### 2.7.2 DPPH scavenging assay

The antioxidant activity was also assessed using the DPPH scavenging assay with slight modifications (Cheng et al., 2022). The sample extract (0.5 mL) was mixed with 6 mL of DPPH solution (0.1 mmol/L) and allowed to react at room temperature for 30 min. The reaction was protected from light. The absorbance of the mixture was measured at 517 nm to determine antioxidant activity. The DPPH radical scavenging activity was calculated as follows:


$$\begin{array}{c} DPPH\;radical\;scavenging\;activity\;\left(\%\right)\\ =\frac{A0-\left(A1-A2\right)}{A0}\;\times \;100\% \end{array}$$

where A_0_ presents the absorbance of the control, A_1_ presents the absorbance of samples (or Vc), and A_2_ presents the absorbance of the blank without DPPH.

### 2.8 CCK-8 assay

Human colorectal cancer cell lines HT-29 and HCT116, as well as the human breast cancer cell line MDA-MB-468, were kindly provided by the Stem Cell Bank of the Chinese Academy of Sciences (Shanghai, China). The colorectal cancer cells were maintained in McCoy’s 5A medium (Gibco™) supplemented with 10% fetal bovine serum (FBS; KM0502, Ozfan, Nanjing, Jiangsu, China) and 1% penicillin/streptomycin (Beyotime Biotechnology, Shanghai, China). The breast cancer cells were maintained in a dedicated cell culture medium (CM-0290B, Procell, Wuhan, Hubei, China).

Cancer cells in the exponential growth phase were seeded into 96-well plates at a density of 5 × 10^3^ cells per well. After 24 hours, the cells were treated with *S. barbata* extracts at various concentrations: 0 μg/mL, 12.5 μg/mL, 25 μg/mL, 50 μg/mL, 100 μg/mL, 200 μg/mL, 400 μg/mL, 600 μg/mL, and 800 μg/mL. Each concentration had six replicates. Following the 72-hour incubation, 10 μL of Cell Counting Kit-8 (CCK-8) solution was added to each well, and the cells were incubated for an additional 2 hours. The IC_50_ of the extracts was determined by measuring the optical density at 450 nm using a microplate reader and plotting the dose–response curve.

## 3 Results and discussion

### 3.1 Phenotypic changes and biomass accumulation patterns of *S. barbata* at different growth stages

**Figure 1:**
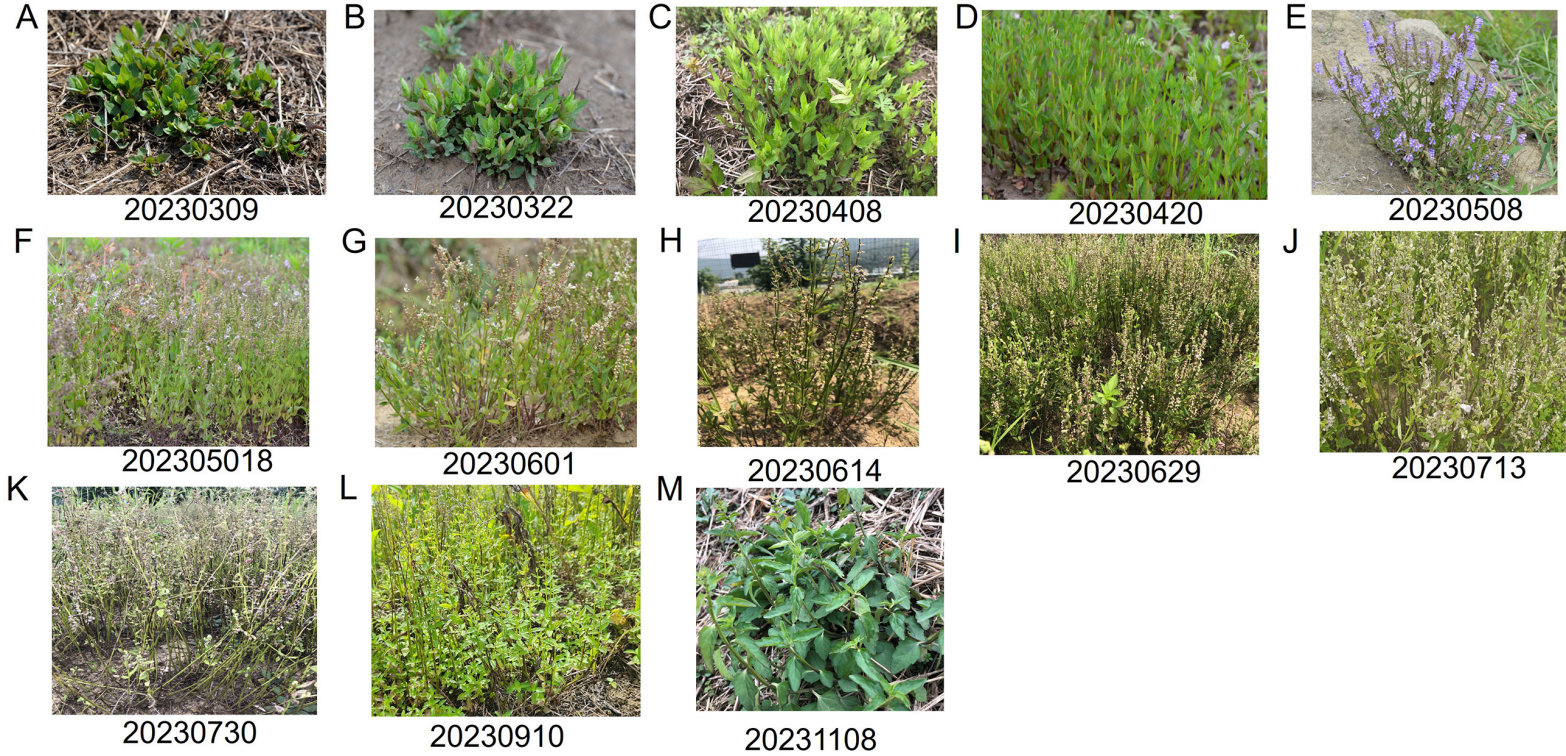
Morphology of the aerial parts of *Scutellaria barbata* at different stages over 1 year. **(A–K)** Morphological changes of the aerial parts of *S. barbata* from greening to withering during the first growth cycle (from March to July). **(L)** Morphology of the aerial parts during the second round of vigorous growth. **(M)** Morphology of the aerial parts during the third round of vigorous growth.

**Figure 2:**
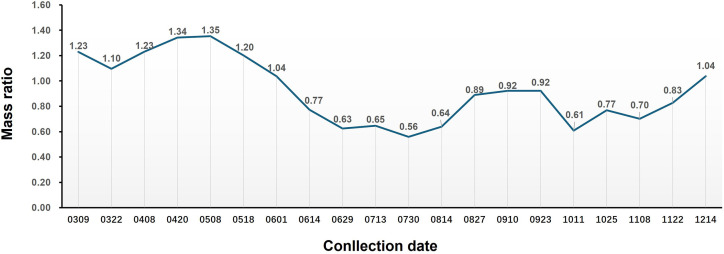
Changes in the mass ratio between the aerial parts and roots of *Scutellaria barbata* at different growth stages.

The phenotypic change in the aerial parts of *S. barbata* and the mass ratio of the aerial part to the root over 1 year are shown in **Figures 1**, **2**, respectively. The aerial parts of *S. barbata* started to grow in early March. From March to early May, the biomass ratio of the aerial part to the root gradually increased, indicating vigorous growth and rapid biomass accumulation in the aerial parts during this time. However, from May onward, this ratio began to gradually decrease. Through observation, it was discovered that *S. barbata* entered the flowering period and began reproductive growth at this time, and after that, the aerial parts gradually withered. At the beginning of August, the aboveground parts underwent a second round of vigorous growth. By mid-October, they withered again. Although the aerial parts of *S. barbata* exhibited regrowth in November and December, the biomass accumulation during this time significantly slowed down compared to that in spring.

### 3.2 Comparative analysis of the spatial distribution of metabolites in *S. barbata*

**Figure 3:**
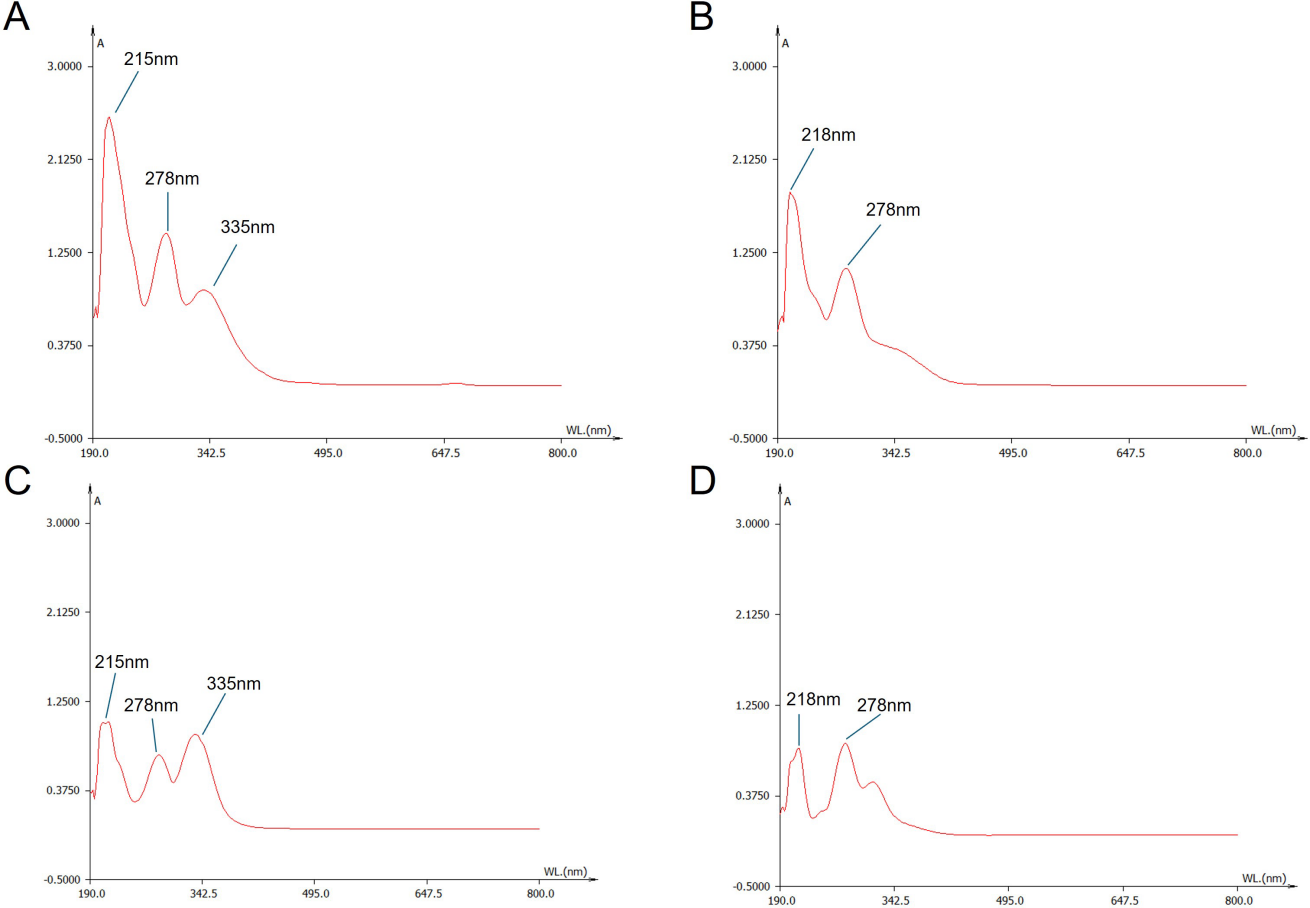
UV–Visible wavelength spectra of *Scutellaria barbata* extracts and representative flavonoid compounds. **(A)** Spectrum of the aerial part extract of *S. barbata*. **(B)** Spectrum of the root extract of *S. barbata*. **(C)** Spectrum of scutellarin. **(D)** Spectrum of baicalin.

Previous studies have isolated and identified various chemical components in *S. barbata*, but the differences in chemical composition between its aerial parts and roots remain unclear (Wang et al., 2020). In this study, UV–VIS spectroscopy was employed to scan the extract solutions of both parts. As illustrated in **Figure 3**, the aerial extract exhibited three distinct absorption peaks at 215 nm, 278 nm, and 335 nm, while the root extract showed only two prominent peaks at 218 nm and 278 nm. These differences in UV absorption spectra suggested that the chemical compositions of the aerial parts and roots of *S. barbata* were distinct.

**Figure 4:**
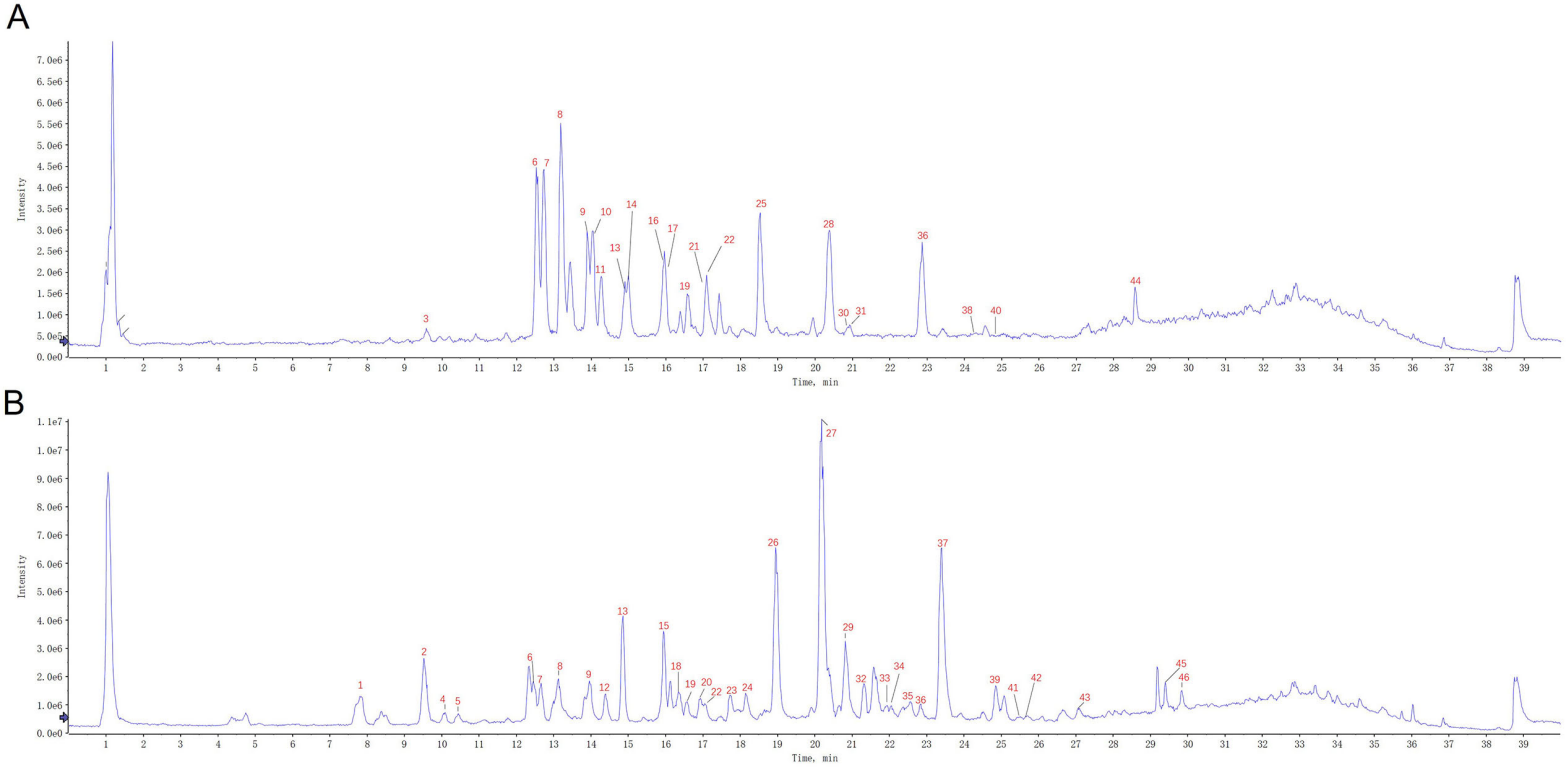
The representative TIC of *Scutellaria barbata* extracts on UHPLC–QTOF–MS. **(A)** Aerial part of *S. barbata*. **(B)** Root of *S. barbata*. The peak numbers in the TIC graph are consistent with those in **Table 1**. TIC, total ion chromatogram; UHPLC–QTOF–MS, ultra-high-performance liquid chromatography–quadrupole time-of-flight–mass spectrometry.

**Figure 5:**
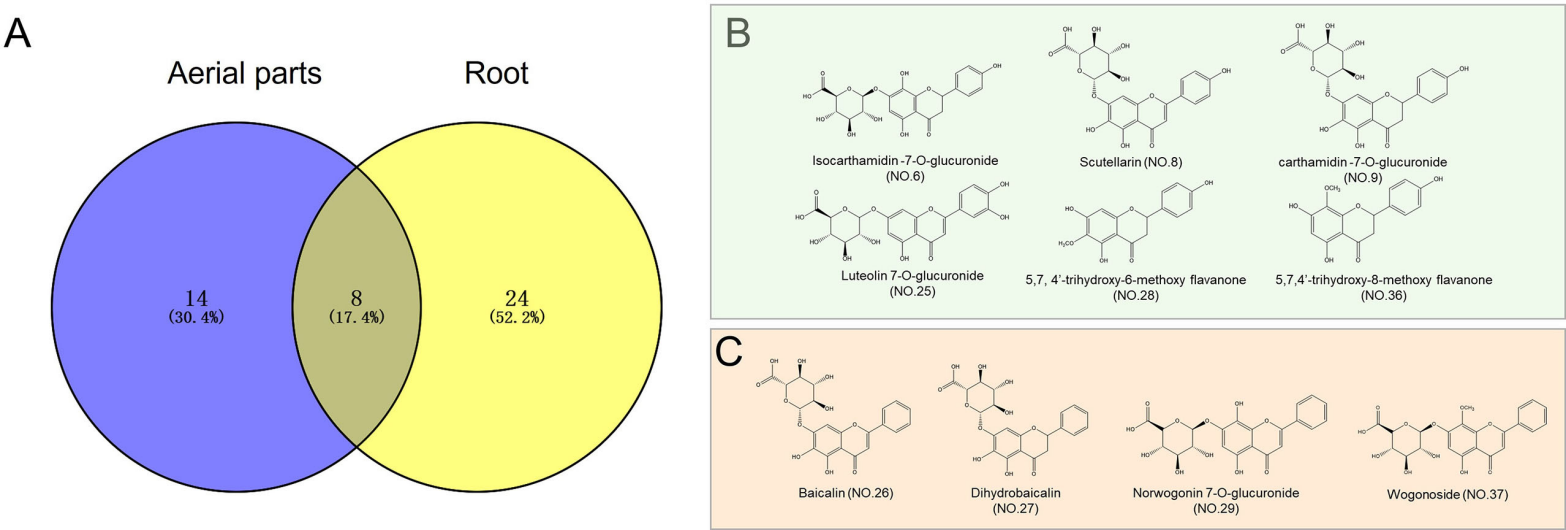
Distribution of the identified compounds in *Scutellaria barbata* and the chemical structures of representative compounds. **(A)** Venn diagram showing the distribution of identified compounds in *S. barbata*. **(B)** Chemical structures of the main flavonoids identified in the aerial parts of *S. barbata*. **(C)** Chemical structures of the main flavonoids identified in the roots of *S. barbata*.

HPLC–QTOF–MS/MS was also employed to elucidate the differences in chemical profiles between the aerial parts and roots of *S. barbata*. There were marked differences in the total ion chromatograms (TICs) between the aerial parts and roots, indicating significant variations in their chemical compositions, consistent with the UV–VIS spectroscopy results (**Figure 4**). Upon analyzing the mass spectrometry data, a total of 46 compounds were identified in both parts of *S. barbata* (**Table 1**), mainly flavonoids and phenylethanol glycosides (Nos. 13 and 15). Among these, eight compounds were common to both the aerial parts and roots, including isocarthamidin-7-*O*-glucuronide, carthamidin-7-*O*-glucuronide, scutellarin, acteoside, 5,7,4′-trihydroxy-6-methoxy flavanone, 5,7,4′-trihydroxy-8-methoxy flavanone, isomer of isocarthamidin-7-*O*-glucuronide, and isomer of carthamidin-7-*O*-glucuronide. Fourteen compounds were found exclusively in the aerial parts, while 24 compounds were detected only in the roots of *S. barbata* (**Figure 5A**, **Table 1**). As depicted in **Figures 5B, C**, the aerial parts of *S. barbata* mainly accumulated 4′-hydroxyflavones such as scutellarin and luteolin 7-*O*-glucuronide, whereas the roots primarily accumulated specialized flavonoids lacking a 4′-OH group on their B-rings, such as baicalin and wogonoside. Similarly, our previous study revealed that the roots of *Scutellaria baicalensis* also accumulated large amounts of flavonoids without the 4′-OH group (Xu et al., 2018b). The flavonoid distribution in the aerial parts and roots of *S. barbata* is similar to the spatial distribution patterns of flavones in *S. baicalensis*. Research has shown that *S. baicalensis* possesses an evolved pathway for the biosynthesis of specific, bioactive 4′-deoxyflavones in its roots, involving enzymes such as flavone synthase II-2, cinnamic acid-specific coenzyme A ligase-7, and chalcone synthase-2 (Zhao et al., 2016). These genes are highly expressed in the roots of *S. baicalensis*, leading to the accumulation of flavonoids lacking hydroxylation at the 4′ position of the B-ring. Since *S. barbata* and *S. baicalensis* are closely related species in the same genus, it is likely that a similar flavonoid without a 4′-OH group biosynthetic pathway exists in the roots of *S. barbata*. This pathway may run parallel to the classic flavone biosynthesis and specifically produce flavonoids that lack 4′-OH groups.

**Table 1:** Characterization of chemical constituents in *Scutellaria barbata* by UHPLC–QTOF–MS/MS.

No.	t_R_ (min)	Molecular formulas	[M–H] predicted	[M–H]^−^ measured (error)	(−)-ESI–MS/MS (*m*/*z*)	[M+H]^+^ measured (error)	Identification
1	7.79	C_21_H_20_O_13_	479.0831	479.0809 (−4.6)	303.0499, 285.0389, 217.0498, 193.0352, 161.0250, 139.0050, 123.0107	481.0971 (−1.2)	5,6,7,3′,4′-Pentahydroxy flavanone-7-*O*-glucuronide ^B^
2	9.421	C_21_H_16_O_13_	475.0518	475.0495 (−4.9)	299.0192, 271.0237, 227.0361, 213.0227, 201.0202	477.0663 (−0.1)	5,6,7-Trihydroxy-8-methoxy flavone-7-*O*-β-d-glucuronide ^B^
3	9.64	C_27_H_30_O_15_	593.1512	593.149 (−3.7)	575.1407, 503.1181, 473.1066, 383.0751, 353.0652	595.1658 (0.1)	Vicenin-2 ^A^
4	10.102	C_21_H_20_O_13_	479.0831	479.0805 (−5.5)	303.0502, 285.0397, 217.0516, 193.0355, 161.0256, 139.0053, 123.0103	481.0973 (−0.8)	Isomer of 5,6,7,3′,4′-pentahydroxy flavanone-7-*O*-glucuronide ^B^
5	10.458	C_21_H_20_O_13_	479.0831	479.0805 (−5.5)	303.0501, 285.0378, 217.0439, 193.0348, 161.0249, 139.0053,	481.0975 (−0.3)	Isomer of 5,6,7,3′,4′-pentahydroxy flavanone-7-*O*-glucuronide ^B^
6	12.44	C_21_H_20_O_12_	463.0882	463.0868 (−3.0)	287.0551, 269.0453,259.0609,181.0152, 166.9997, 153.0210,139.0060, 119.0532,113.0284	465.1024 (−0.8)	Isocarthamidin-7-*O*-glucuronide ^AB^
7	12.626	C_21_H_20_O_12_	463.0882	463.087 (−2.6)	287.0550, 269.0458, 259.0610, 193.0166, 181.0154, 166.9998, 153.0210, 119.0535,113.0277	465.1026 (−0.3)	Isomer of isocarthamidin-7-*O*-glucuronide ^AB^
8	13.167	C_21_H_18_O_12_	461.0725	461.0716 (−2.1)	285.0401, 267.0301, 257.0386, 239.0342, 213.0551, 195.0455, 175.0261, 113.0279	463.0873 (0.4)	Scutellarin ^AB^
9	13.909	C_21_H_20_O_12_	463.0882	463.0865 (−3.7)	287.0548, 269.0452, 259.0615, 193.0141, 181.0147, 166.9990, 153.0202, 139.0052,119.0521, 113.0274	465.1027 (−0.1)	Carthamidin-7-*O*-glucuronide ^AB^
10	14.01	C_21_H_20_O_12_	463.0882	463.0866 (−3.5)	287.0546, 269.0449, 259.0601, 193.0341, 181.0151, 166.9995, 153.0205, 139.0053, 119.0532, 113.0277	465.1026 (−0.3)	Isomer of carthamidin-7-*O*-glucuronide ^A^
11	14.246	C_21_H_20_O_10_	431.0984	431.0966 (−4.1)	26930446, 225.0561, 117.0372	433.1133 (0.9)	Apigenin-7-*O*-glucoside ^A^
12	14.384	C_21_H_18_O_12_	461.0725	461.0702 (−5.1)	285.0389, 267.0280, 223.0389, 185.0628, 139.0045, 113.0270	463.0873 (0.4)	Isomer of scutellarin ^B^
13	14.883	C_29_H_36_O_15_	623.1981	623.1958 (−3.6)	623.1942, 461.1644, 161.0255, 135.0495	625.2129 (0.3)	Acteoside ^AB^
14	14.976	C_21_H_18_O_12_	461.0725	461.0706 (−4.2)	285.0400, 257.0411, 241.0510, 229.0490, 213.0549, 185.0690, 113.0268	463.0871 (0)	Kaempferol-3-*O*-glucuronide ^A^
15	15.942	C_29_H_36_O_15_	623.1981	623.1955 (−4.2)	461.1645, 161.0265, 133.0310	625.2119 (−1.3)	Isomer of acteoside ^B^
16	15.973	C_21_H_20_O_11_	447.0933	447.0922 (−2.4)	271.0611, 175.0266, 151.0051, 113.0277	449.1077 (−0.3)	Naringenin-7-*O*-glucuronide ^A^
17	15.973	C_21_H_18_O_11_	445.0776	445.0762 (−3.2)	269.0455, 225.0572, 175.0254, 113.0278	447.0922 (0)	Apigenin-7-*O*-glucuronide ^A^
18	16.37	C_21_H_20_O_12_	463.0882	463.0862 (−4.3)	301.0349, 287.0549, 239.0369, 166.9981, 139.0052	465.1026 (−0.3)	Isomer of Isocarthamidin-7-*O*-glucuronide ^B^
19	16.61	C_22_H_20_O_12_	475.0882	475.087 (−2.5)	299.0556, 284.0323, 175.0253	477.1022 (−1.2)	5,7,8-Trihydroxy-6-methoxy flavone-7-*O*-glucuronide ^AB^
20	16.899	C_21_H_18_O_12_	461.0725	461.0704 (−4.7)	285.0395, 267.0280, 239.0351, 223.0410, 167.0008, 151.0055, 139.0050, 113.0260	463.0869 (−0.4)	Isoscutellarein-7-*O*-β-d-glucuronide ^B^
21	17.101	C_22_H_20_O_12_	475.0882	475.0866 (−3.4)	299.0548, 284.0318, 175.0249, 113.0271	477.1029 (0.3)	5,7,2′-Trihydroxy-6-methoxy flavone-7-*O*-glucuronide ^A^
22	17.101	C_22_H_22_O_12_	477.1039	477.1022 (−3.5)	301.0710, 286.0479, 181.0143, 175.0258, 165.9919, 113.0276	479.1180 (−0.8)	5,7,2′-Trihydroxy-8-methoxy flavanone-7-*O*-glucuronide ^AB^
23	17.733	C_22_H_22_O_11_	461.1089	461.1067 (−4.8)	299.0550, 284.0323, 165.9916	463.1237 (0.5)	5,7,2′-Trihydroxy-6-methoxy flavone 7-*O*-glucoside ^B^
24	18.154	C_22_H_20_O_12_	475.0882	475.0858 (−5.1)	299.0552, 284.0318, 175.0256, 165.9915, 113.0276	477.1021 (−1.4)	5,7,8-Trihydroxy-6-methoxy flavone 7-*O*-glucuronide ^B^
25	18.549	C_21_H_18_O_12_	461.0725	461.0705 (−4.4)	285.0397, 257.0448, 241.0498, 229.0489, 213.0570, 197.0616, 187.0407, 175.0261, 171.0465, 113.0267	463.0807 (−0.2)	Luteolin 7-*O*-glucuronide ^A^
26	18.96	C_21_H_18_O_11_	445.0776	445.0754 (−5.0)	269.0441, 251.0339, 241.0475, 223.0394, 195.0453, 113.0277	447.0923 (0.3)	Baicalin ^B^
27	20.179	C_21_H_20_O_11_	447.0933	447.0914 (−4.2)	271.0598, 253.0502, 243.0666, 113.0280	449.1075 (−0.8)	Dihydrobaicalin ^B^
28	20.374	C_16_H_14_O_6_	301.0718	301.0714 (−1.2)	286.0476, 258.0520, 181.0146, 165.9917, 137.9976, 119.0531, 110.0045	303.0864 (0.3)	5,7,4′-Trihydroxy-6-methoxy flavanone ^A^
29	20.81	C_21_H_18_O_11_	445.0776	445.0758 (−4.1)	269.0450, 241.0501, 225.0554, 213.0550, 197.0616, 171.0465, 113.0272	447.0922 (0)	Norwogonin 7-*O*-glucuronide ^B^
30	20.9	C_15_H_10_O_6_	285.0405	285.04 (−1.6)	257.0396, 241.0500, 217.0496, 199.0410, 175.0405, 151.0042, 133.0314	287.0549 (−0.4)	Luteolin ^A^
31	20.938	C_31_H_40_O_15_	651.2294	651.2285 (−1.5)	475.1829, 329.1212, 193.0497, 175.0400, 160.0157	653.2431 (−1.4)	Cistanoside D ^A^
32	21.276	C_21_H_18_O_11_	445.0776	445.0757 (−4.3)	269.0443, 241.0496, 225.0538, 197.0606, 171.0456	447.0921 (−0.2)	Norwogonin 8-*O*-glucuronide ^B^
33	21.925	C_21_H_18_O_10_	429.0827	429.0809 (−4.2)	253.0500, 225.0543, 209.0611, 175.0254, 143.0530, 113.0278	431.0972 (−0.2)	Chrysin 7-*O*-glucuronide ^B^
34	22.074	C_22_H_20_O_11_	459.0933	459.0916 (−3.7)	283.0609, 268.0375, 175.0252, 113.0280	461.1077 (−0.3)	Oroxylin A 7-*O*-glucuronide ^B^
35	22.578	C_23_H_24_O_11_	475.12459	475.1224 (−4.6)	313.0702, 289.0467, 283.0236, 255.0299	477.1387 (−0.9)	Cirsimaritin 4′-glucoside ^B^
36	22.88	C_16_H_14_O_6_	301.0718	301.0714 (−1.2)	286.0475, 258.00513, 181.0147, 165.9918, 137.9979, 119.0536, 110.0052	303.0863 (0)	5,7,4′-Trihydroxy-8-methoxy flavanone ^AB^
37	23.402	C_22_H_20_O_11_	459.0933	459.0919 (−3.0)	283.0601, 268.0368, 175.0261, 113.0281	461.1074 (−1)	Wogonoside ^B^
38	24.327	C_16_H_12_O_6_	299.0561	299.0559 (−0.7)	284.0320, 256.0367, 227.0331, 212.0467, 136.9891	301.0712 (1.8)	4′-Hydroxywogonin ^A^
39	24.844	C_23_H_22_O_12_	489.1039	489.1022 (−3.4)	313.0711, 298.0474, 283.0240, 255.0306, 175.0252, 113.0280	491.1182 (−0.4)	5,7-Dihydroxy-8,2′-dimethoxy flavone 7-*O*-glucuronide ^B^
40	24.928	C_16_H_12_O_6_	299.0561	299.0557 (−1.4)	284.0316, 256.0349, 227.0334, 212.0468, 136.9899	301.0707 (0.1)	Hispidulin ^A^
41	25.477	C_15_H_10_O_5_	269.0456	269.0455 (0.2)	241.0508, 225.0548, 213.0562, 197.0611, 171.0463, 155.0519	271.0599 (−0.7)	Apigenin ^B^
42	25.703	C_16_H_14_O_6_	301.0718	301.0712 (−1.9)	286.0461, 195.0296, 181.0144, 165.9918, 155.0360, 140.0128, 119.0518, 110.0015	303.0862 (−0.4)	Isomer of 5,7,4′-trihydroxy-8-methoxy flavanone ^B^
43	27.096	C_15_H_10_O_5_	269.0456	269.045 (−2.0)	251.0325, 241.0492, 223.0401, 195.0444, 169.0667, 139.0043	271.0600 (−0.4)	Baicalin ^B^
44	28.575	C_15_H_12_O_5_	271.0612	271.0613 (0.4)	187.0413, 119.0534	273.0758 (0.2)	Naringenin ^A^
45	29.405	C_16_H_12_O_5_	283.0612	283.0611 (−0.3)	268.0366, 239.0349, 211.0394, 184.0534, 163.0051, 110.0041	285.0763 (1.9)	Wogonin ^B^
46	29.838	C_17_H_14_O_6_	313.0718	313.0715 (−0.8)	298.0482, 283.0245, 255.0301, 183.0454, 164.9840	315.0864 (0.3)	5,7-Dihydroxy-6,8-dimethoxy flavone ^B^

UHPLC–QTOF–MS, ultra-high-performance liquid chromatography–quadrupole time-of-flight–mass spectrometry; ESI–MS/MS, electrospray ionization–tandem mass spectrometry.

^A^Compound identified in the aerial parts of *S. barbata*.

^B^Compound identified in the roots of *S. barbata*. The identification of compounds No. 3 and No. 40 were based on Li et al. (2015), and the other compounds were identified according to Xu et al. (2018b).

### 3.3 Seasonal dynamics of quality-related flavonoids in *S. barbata*

**Figure 6:**
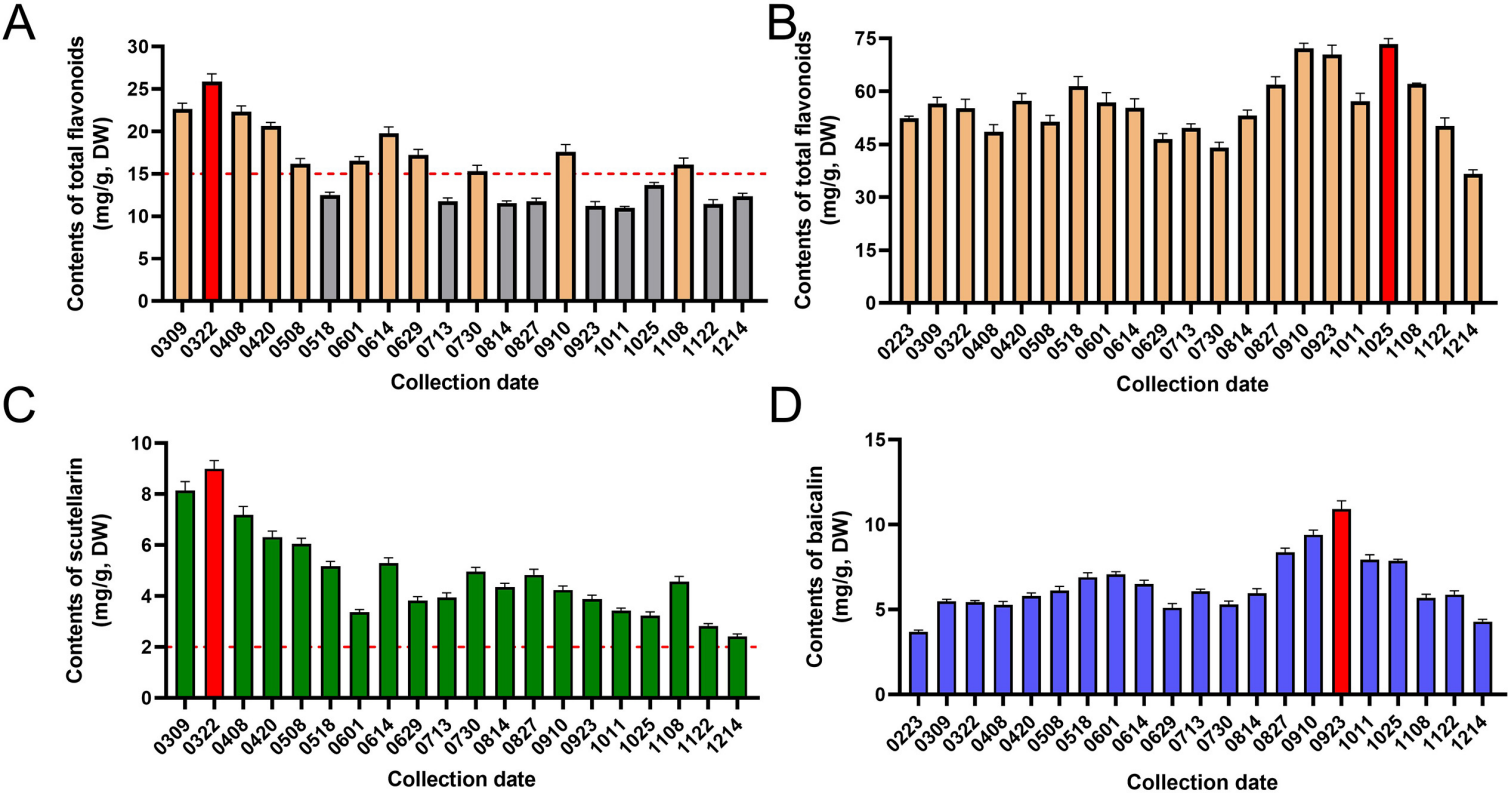
The accumulation patterns of quality-related flavonoids in *Scutellaria barbata*. **(A)** The contents of total flavonoids in aerial parts of *S. barbata* at different collection dates. **(B)** The contents of total flavonoids in roots of *S. barbata* at different collection dates. **(C)** The contents of scutellarin in aerial parts of *S. barbata* at different collection dates. **(D)** The contents of baicalin in roots of *S. barbata* at different collection dates.

The accumulation of total flavonoids in the aerial parts and roots of *S. barbata* was quantitatively analyzed at different periods over 1 year, as shown in **Figures 6A, B**. The total flavonoid content in the aerial parts ranged from 10.99 ± 0.15 mg/g (collected on October 11) to 25.87 ± 0.91 mg/g (collected on March 22), while in the roots, it varied from 36.65 ± 1.12 mg/g (collected on December 14) to 73.38 ± 1.56 mg/g (collected on October 25). The accumulation of flavonoids in the aerial parts varied significantly across different growth stages. From early March to late April, when *S. barbata* primarily engaged in vegetative growth, the flavonoid content in the aerial parts was high and relatively stable. During May, the flowering period, there was a rapid decrease in flavonoid accumulation in the aerial parts. This may be due to the anthocyanidins and other flavonoids derived from the common biosynthetic pathway and share the same precursors (Li et al., 2016). The inhibition of flavonoid synthesis in vegetative organs leads to an increased accumulation of anthocyanidins in reproductive organs (Lim et al., 2016). From June to August, as the aboveground parts began to wither, the contents of flavonoids initially increased and subsequently decreased. In early September and early November, the aboveground parts regrew, and flavonoid accumulation exhibited a similar pattern. However, the peak levels during these later stages (samples from September 10 and November 8) were lower than those observed during the initial vegetative growth phase (sample from March 22).

Changes in flavonoid accumulation may be related to the intensity and duration of daylight. Light, particularly UV radiation, can cause DNA damage and other cellular injuries in plants (Banerjee et al., 2024). Flavonoids act as effective UV filters, protecting plant cells by absorbing and dissipating UV radiation (Hichri et al., 2011; Ferreyra et al., 2021; Cao et al., 2024). Numerous studies have shown that UV radiation is a key environmental signal that triggers flavonoid synthesis in plants (Liu et al., 2015; Henry-Kirk et al., 2018; Xie et al., 2020). Plants grown under high-light conditions or longer daylight periods typically accumulated more flavonoids compared to those grown in shaded or low-light environments (Götz et al., 2010; Deng et al., 2012). *S. barbata* primarily grows from March to August, during the spring and summer months. The high abundance of flavonoids during this period likely helps protect the leaves against the increasing light duration and UV intensity (Brunetti et al., 2013). In contrast, the subsequent greening periods in September and November, which occur during autumn and winter, coincide with shorter days and lower light intensity, reducing the need for UV protection. This seasonal variation may explain the differences in flavonoid accumulation observed during the vegetative growth stages of *S. barbata* at different times over 1 year.

Scutellarin is a quality marker for *S. barbata* as stipulated in the Chinese Pharmacopoeia. This study further analyzed the accumulation pattern of scutellarin in aerial parts of *S. barbata* across different growth stages. As shown in **Figure 6C**, the content of scutellarin in the aerial parts ranged from 2.41 ± 0.10 mg/g (collected on December 14) to 9.00 ± 0.32 mg/g (collected on March 22). The accumulation pattern of scutellarin in the aerial parts of *S. barbata* was consistent with that of total flavonoid. Scutellarin levels peaked in late March and gradually declined as the plant entered its flowering period. Following the end of reproductive growth, the content of scutellarin in *S. barbata* increased again, reaching another peak in August. The results indicated that the scutellarin content in the aerial parts of *S. barbata* exceeded the Chinese Pharmacopoeia’s requirement (not less than 0.20%) at almost all development stages. However, the total flavonoid content in *S. barbata* meets the Chinese Pharmacopoeia standards (not less than 1.50%) only when the aerial parts grow vigorously.

This study found that the total flavonoid content in the roots of *S. barbata* ranged from 36.65 ± 1.12 mg/g (collected on December 14) to 73.38 ± 1.56 mg/g (collected on October 25). The accumulation pattern of total flavonoids in the roots differed significantly from that in the aerial parts. From February to July, while the total flavonoid content in the aerial parts underwent dramatic changes, the content in the roots only fluctuated slightly. However, from August to December, the accumulation of total flavonoids in the roots underwent two times distinct increases. This metabolic shift may be a response to environmental stress through nutrient reallocation. Reduced light hours, decreasing temperatures, and decreased water availability are common in autumn. In roots, flavonoids can stabilize cell membranes and mitigate oxidative stress induced by low temperatures, aiding in cold acclimation (Genzel et al., 2021; Song et al., 2023). The accumulated flavonoids may also improve drought resistance by maintaining cellular osmotic balance and protecting cells from dehydration (Nakabayashi et al., 2014; Zuo et al., 2024). Additionally, during the withering period, plants undergo metabolic reconstruction, leading to the accumulation of secondary metabolites in the roots (Zeng et al., 2017; Liu et al., 2020). These factors may contribute to the accumulation of total flavonoids in the roots of *S. barbata* in the fall.

The chemical profiles of *S. barbata* roots and aerial parts differ significantly, yet the Chinese Pharmacopoeia does not define specific chemical components for assessing the quality of roots. In this study, baicalin (No. 26 in **Figure 4B**) was identified as a major component in the roots of *S. barbata*. Previous studies have indicated that baicalin can exhibit significant pharmacological activity and serve as a quality marker for *S. baicalensis*, a root-based Chinese medicinal material (Zhang et al., 2023; Wang et al., 2024; Chinese Pharmacopoeia Commission, 2020). Therefore, the accumulation pattern of baicalin in the roots was examined to gain insights into the quality changes of *S. barbata*. The content of baicalin in *S. barbata* roots varied from 4.29 ± 0.14 mg/g (collected on December 14) to 10.92 ± 0.48 mg/g (collected on September 23). **Figure 6D** illustrates that the accumulation pattern of baicalin in the roots of *S. barbata* was inversely related to that of scutellarin in the aerial parts. The scutellarin was primarily accumulated during the vigorous growth of the aerial parts in spring, while baicalin in the roots of *S. barbata* reached the highest accumulation level when the aerial parts grew slowly in autumn.

The findings indicated that the aerial parts of *S. barbata* could be harvested several times annually. To enhance biomass accumulation and comply with the Chinese Pharmacopoeia’s standards for total flavonoid and scutellarin content, it is recommended to harvest the aerial parts of *S. barbata* during the vigorous growth periods in March, April, June, early September, and early November. When roots are needed, harvesting should take place in the autumn season from August to November to maximize the flavonoid accumulation in the roots of *S. barbata*.

### 3.4 Comparative analysis of the pharmacodynamic activities of the aerial parts and roots of *S. barbata*

**Figure 7:**
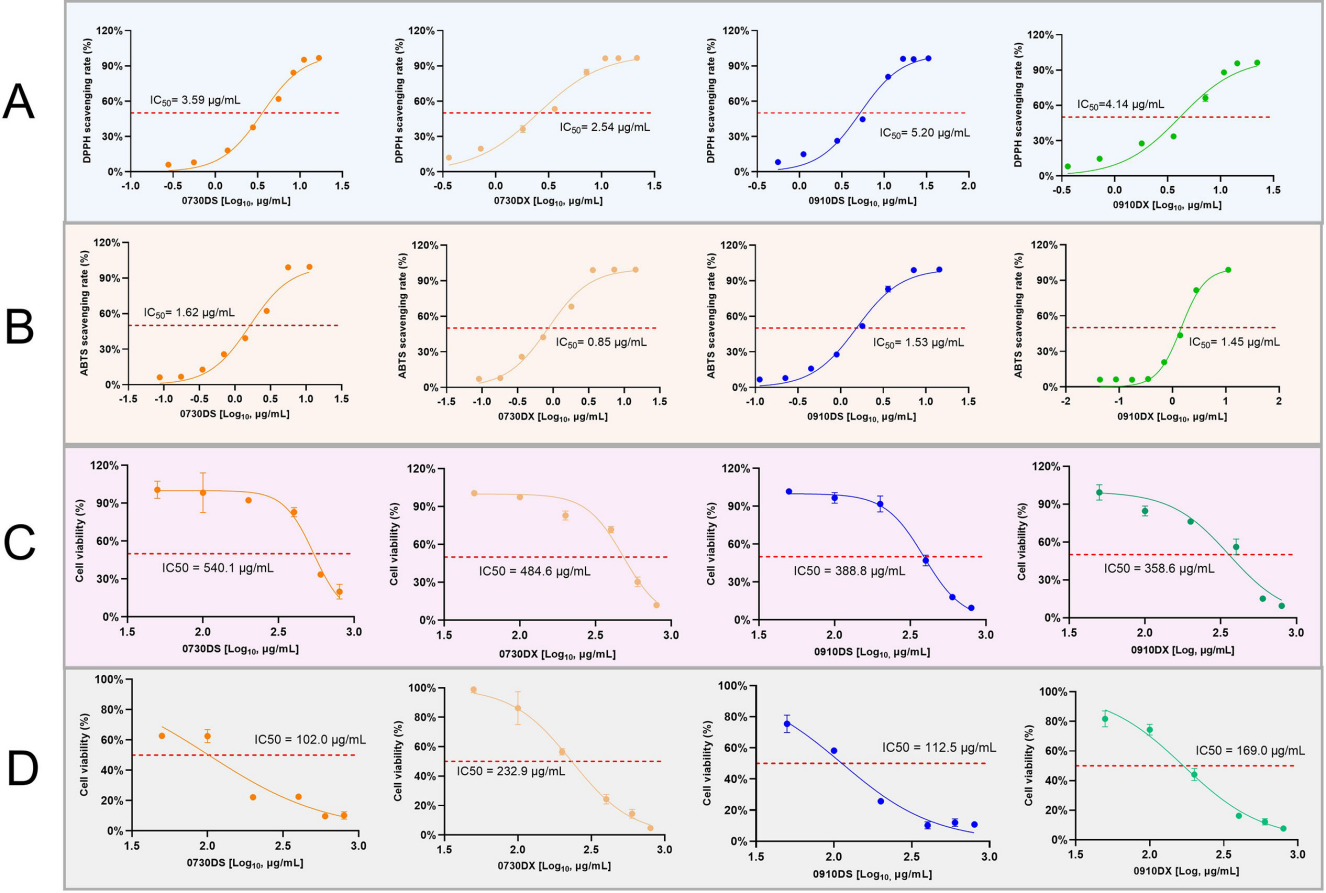
Comparative activities of extracts from the aerial parts and roots of *Scutellaria barbata*. **(A)** DPPH free radical scavenging activity. **(B)** ABTS free radical scavenging activity. **(C)** Inhibitory activity on breast cancer cell proliferation. **(D)** Inhibitory activity on colon cancer cell proliferation. 0730, sample collected on July 30; 0910, sample collected on September 10; DS, aerial part extract of *S. barbata*; DX, root extract of *S. barbata*.

We observed distinct differences in the chemical profiles of the aerial and underground parts of *S. barbata*, but the variation in their pharmacodynamic activities remained unclear. Previous studies have demonstrated that *S. barbata* extracts exhibit strong antioxidant activity and inhibit cancer cell proliferation (Chen et al., 2020; Liu et al., 2022). Therefore, a functional analysis was performed to assess the *in vitro* antioxidant and cytotoxic activities of the different parts of *S. barbata*. **Figures 7A, B** illustrate that both the aerial parts extracts (DS) and root extracts (DX) of *S. barbata* exhibited good antioxidant activity. This activity was concentration-dependent with the total flavonoids in the extracts. The DX demonstrated stronger free radical scavenging activity than that of DS, regardless of whether assessed using the DPPH or ABTS method. To evaluate the differences in the effects of DS and DX on cancer cell proliferation, CCK-8 assays were performed. HT-29 and MDA-MB-468 cells were treated with DS and DX, respectively, resulting in a dose-dependent decrease in cell viability (**Figures 7C, D**). The IC_50_ of DX in MDA-MB-468 cells was lower than that of DS. However, DS exhibited a greater cytotoxic activity than DX in HT-29 cells.

The biological activity of a compound is closely linked to its structure. While flavonoids share a similar core structure, their activities differ due to variations in the types, numbers, and positions of functional groups on core structures. In this study, we found that although both the aerial parts and roots of *S. barbata* were rich in flavonoids, the type and concentration of these flavonoids differ significantly. The primary flavonoids in the aerial parts were isocarthamidin-7-*O*-glucuronide, scutellarin, carthamidin-7-*O*-glucuronide, luteolin-7-*O*-glucuronide, 5,7,4′-trihydroxy-6-methoxy flavanone, 5,7,4′-trihydroxy-8-methoxy flavanone, while those in the roots were predominantly baicalin, dihydrobaicalin, norwogonin-7-*O*-glucuronide, and wogonoside. Research suggests that the hydroxyl group on the B-ring (C-4′) is more effective for enzyme inhibition, while the hydroxyl group on the A-ring (C-6) is more effective for antioxidant activity. Scutellarin and apigenin, which own the hydroxyl group on the B-ring (C-4′), contribute more to overall enzyme inhibition, while baicalin with no hydroxyl group on the B-ring (C-4′) is a key component to the antioxidant activity of *S. baicalensis* extracts (Li et al., 2018). Yang et al. found that, despite owned structural similarity, baicalin and scutellarin promoted glucose disposal in adipocytes through differential regulation of the AKT pathway, with scutellarin showing much more favorable binding energy to Akt (−29.81 kcal/mol) compared to baicalin (4.04 kcal/mol) (Yang et al., 2017). The IC_50_ of baicalin is approximately twice that of scutellarin, suggesting that the 4′-hydroxyl group plays a positive role in urease inhibition (Yu et al., 2015). While these studies do not completely clarify the differences in efficacy between the aerial parts and roots of *S. barbata*, it is evident that their distinct chemical compositions lead to varying biological activities and pharmacological mechanisms. This aligns with the multi-component, multi-target therapeutic mechanisms characteristic of traditional Chinese medicine. Therefore, it is essential to precisely specify the harvesting parts of *S. barbata* and control the proportion of different parts to ensure the quality and consistency of the medicinal material.

## Conclusion

This study elucidated the distinct chemical profiles and accumulation patterns of flavonoids in the aerial parts and roots of *S. barbata*. The findings demonstrated that the aerial parts could be harvested during periods of vigorous growth in spring and early autumn to maximize flavonoid content, while the roots should be harvested in autumn to ensure high flavonoid accumulation. The study also highlighted significant differences in antioxidant and anticancer activities between the aerial parts and roots, likely linked to their unique chemical compositions. These results underscore the importance of specifying harvesting periods and plant parts to ensure consistent quality in *S. barbata*. The insights would contribute to the development of standardized harvesting protocols and quality control measures for *S. barbata*.

## Data Availability

The original contributions presented in the study are included in the article/**Supplementary Material**. Further inquiries can be directed to the corresponding authors.

## Supplementary material

Supplementary Figure 1 Calibration curves for the quantitative analysis of flavonoids in *S. barbata*. **(A)** Calibration curve for the quantification of total flavonoids in the aerial parts of *S. barbata* by UV spectrophotometry. **(B)** Calibration curve for the quantification of total flavonoids in the roots of S. barbata by UV spectrophotometry. **(C)** Calibration curve for the quantification of scutellarin in the aerial parts of *S. barbata* by HPLC. **(D)** Calibration curve for the quantification of baicalin in the roots of *S. barbata* by HPLC.

Supplementary Figure 2 The TIC chromatogram of *S. barbata* extracts on UHPLC-QTOF-MS in positive ion model. **(A)** Aerial part of *S. barbate*. **(B)** root of *S. barbate*.

Supplementary Figure 3 The representative TIC chromatogram of *S. barbata* collected at different date in negative ion model. **(A)** Aerial part of *S. barbate* collected on November 8. **(B)** Aerial part of *S. barbate* collected on April 20. **(C)** Root of *S. barbate* collected on April 20 or November 8. **(D)** Root of *S. barbate* collected on April 20.
