# Supplementary figures and images for: A comparative study of the quality differences and seasonal dynamics of flavonoids between the aerial parts and roots of Scutellaria barbata
Source: Front Plant Sci. 2024 Dec 2;15:1497664. doi: 10.3389/fpls.2024.1497664 (PMC11648313; doi:10.3389/fpls.2024.1497664)

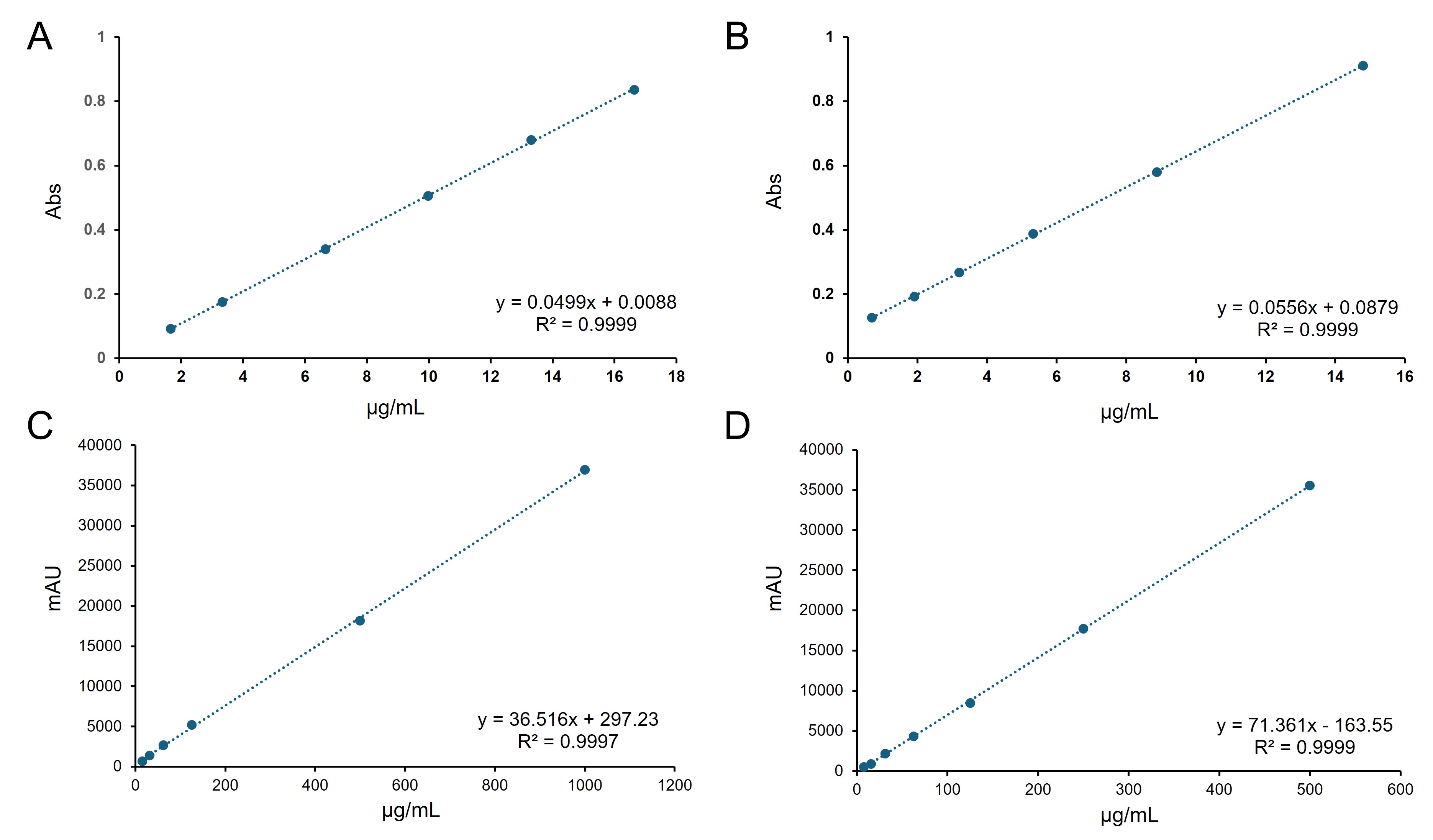

Supplement: Supplementary Figure 1 — Calibration curves for the quantitative analysis of flavonoids in S. barbata. (A) Calibration curve for the quantification of total flavonoids in the aerial parts of S. barbata by UV spectrophotometry. (B) Calibration curve for the quantification of total flavonoids in the roots of S. barbata by UV spectrophotometry. (C) Calibration curve for the quantification of scutellarin in the aerial parts of S. barbata by HPLC. (D) Calibration curve for the quantification of baicalin in the roots of S. barbata by HPLC. [file Image1.jpeg]

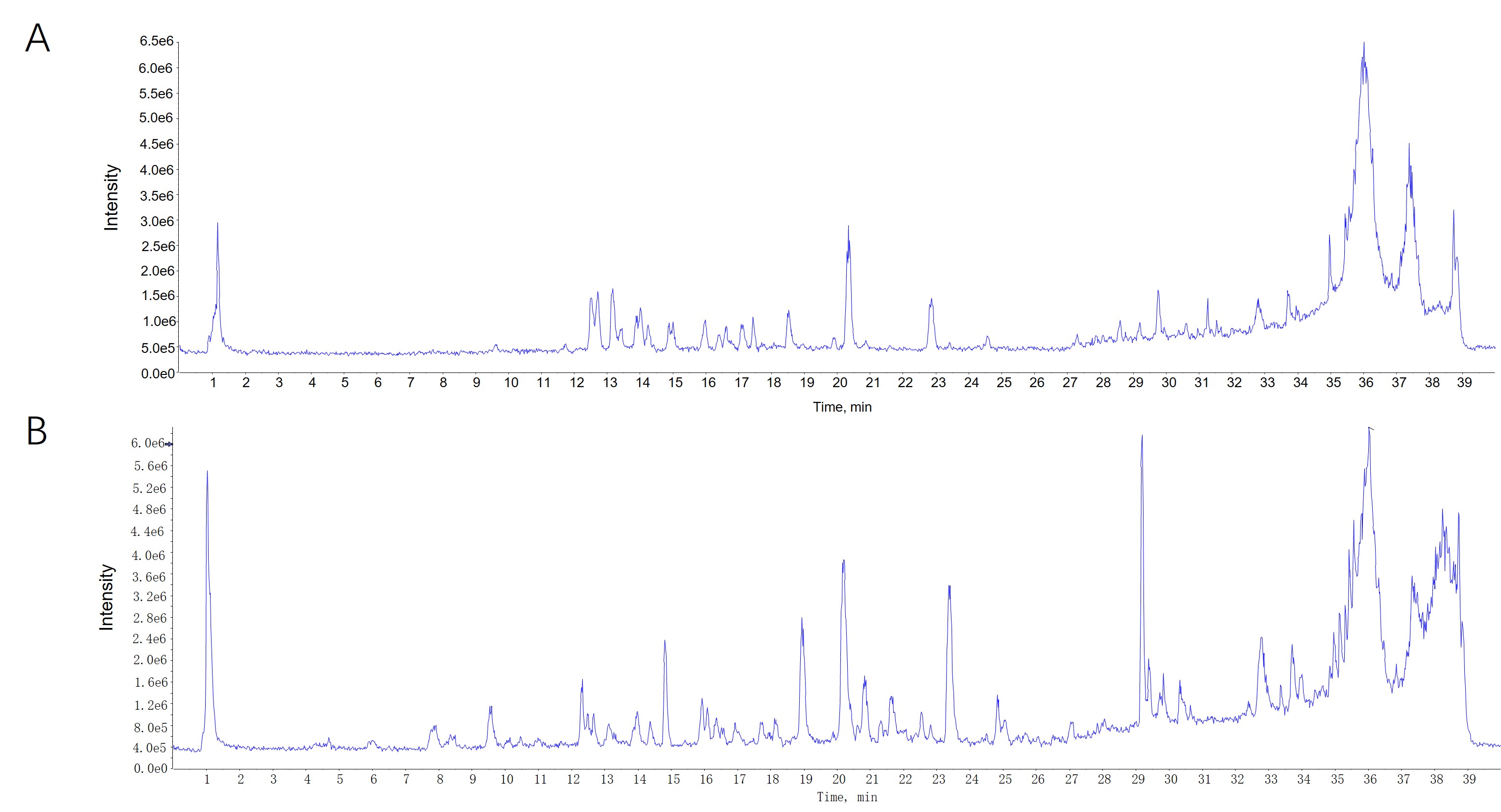

Supplement: Supplementary Figure 2 — The TIC chromatogram of S. barbata extracts on UHPLC-QTOF-MS in positive ion model. (A) Aerial part of S. barbate. (B) root of S. barbate. [file Image2.jpeg]

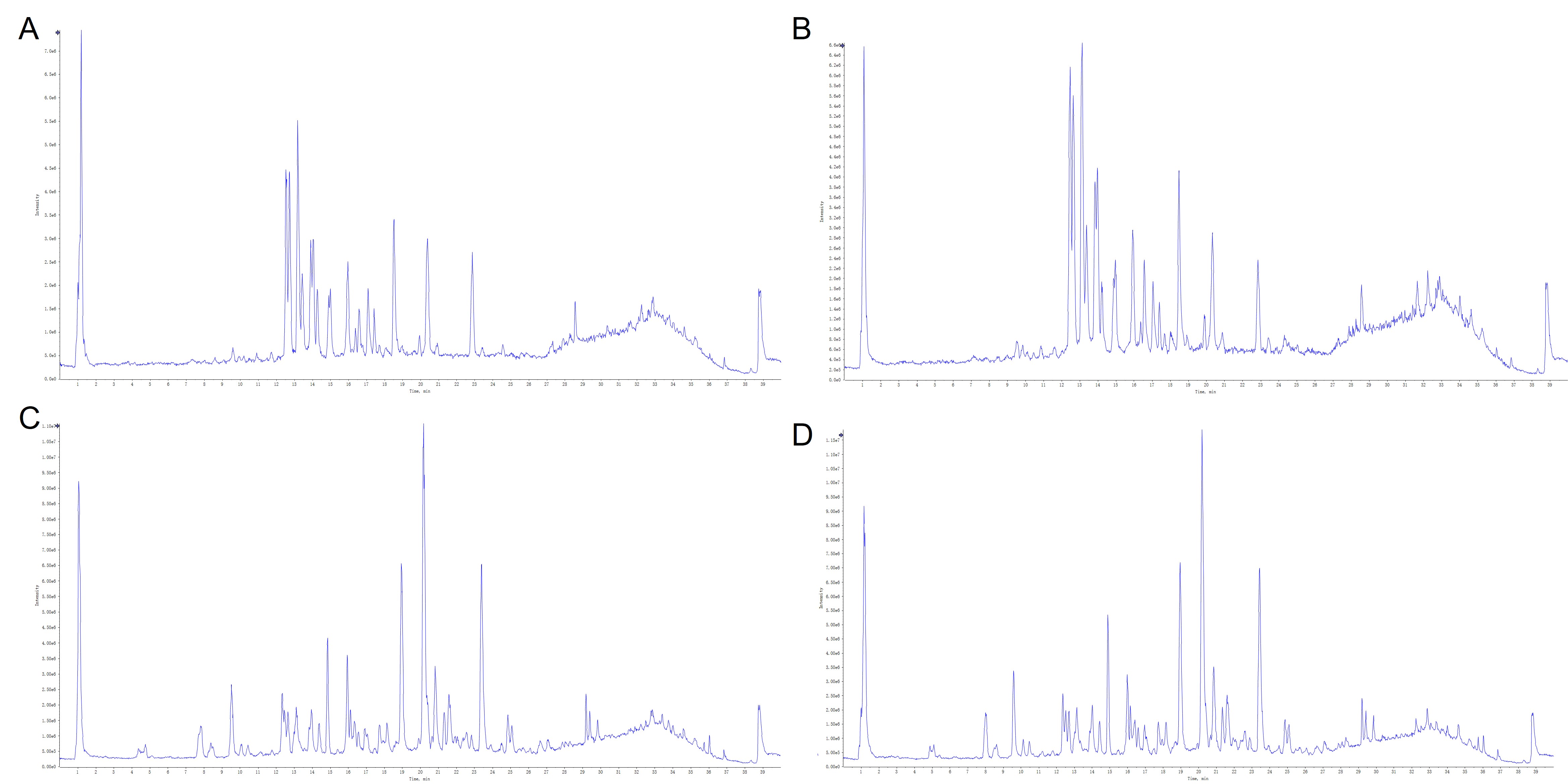

Supplement: Supplementary Figure 3 — The representative TIC chromatogram of S. barbata collected at different date in negative ion model. (A) Aerial part of S. barbate collected on November 8. (B) Aerial part of S. barbate collected on April 20. (C) Root of S. barbate collected on April 20 or November 8. (D) Root of S. barbate collected on April 20. [file Image3.jpeg]
